# Emerging therapies targeting lipoprotein(a): the next frontier in cardiovascular risk reduction

**DOI:** 10.3389/fmed.2025.1727918

**Published:** 2025-12-18

**Authors:** Ibrahim S. Alhomoud

**Affiliations:** Department of Pharmacy Practice, College of Pharmacy, Qassim University, Qassim, Saudi Arabia

**Keywords:** antisense oligonucleotide, atherosclerotic cardiovascular disease, cardiovascular risk, lipoprotein(a), small interfering RNA

## Abstract

Lipoprotein(a) [Lp(a)] is a genetically determined lipoprotein particle composed of apolipoprotein B-100 covalently linked to apolipoprotein(a) [apo(a)] via a disulfide bond. The Lp(a) particle is enriched with oxidized phospholipids (OxPLs), which confer enhanced atherogenic and pro-inflammatory properties compared with low-density lipoprotein (LDL). Robust genetic and epidemiologic evidence demonstrates that elevated Lp(a) levels are independently associated with atherosclerotic cardiovascular disease and calcific aortic valve stenosis. However, no pharmacologic therapy has yet been approved that specifically lower Lp(a) or to demonstrate a reduction in cardiovascular events. Antisense oligonucleotides (e.g., pelacarsen), small-interfering RNAs (e.g., olpasiran, lepodisiran, and zerlasiran), and oral small-molecule Lp(a) inhibitors (e.g., muvalaplin) have demonstrated profound reductions in circulating Lp(a) concentrations, typically achieving decreases of 80–90%. In some studies, the reductions approached or achieved a near-complete suppression. Current genetic and modeling evidence suggests that an absolute reduction of at least 50 mg/dL in Lp(a) levels is required to achieve meaningful cardiovascular benefits. Large-scale outcome trials are now underway to assess the effects of these emerging therapies on cardiovascular and valvular outcomes. Early findings indicate favorable effects on oxidized phospholipids and vascular inflammation, suggesting broader anti-atherogenic potential. As these agents progress toward clinical use, routine Lp(a) measurement and risk stratification will become increasingly essential for personalized cardiovascular prevention. This review summarizes the molecular biology of Lp(a), highlights the limitations of current therapies, and discusses emerging RNA-based and small-molecule approaches with the potential to redefine the management of residual cardiovascular risk.

## 1 Structure and biochemistry of Lp(a)

**Figure 1:**
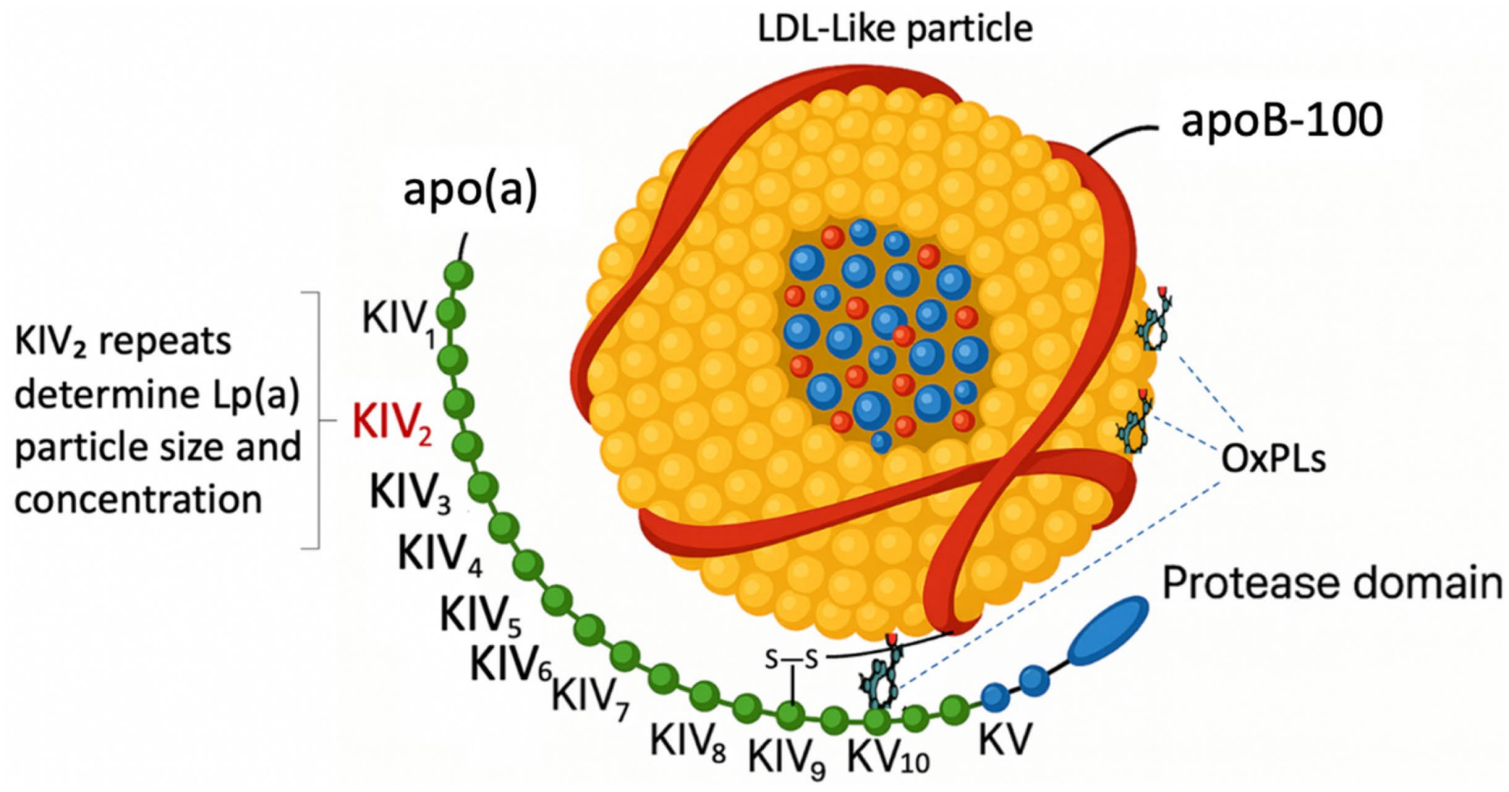
Structure of Lp(a). apo(a), apolipoprotein (a); apo(B), apolipoprotein B; KIV, Kringle IV domain; LDL, low-density lipoprotein; Lp(a), lipoprotein(a); OxPLs, oxidized phospholipids.

Lp(a) is an LDL-like particle composed of apolipoprotein B-100 (apoB-100) covalently linked to apo(a) via a disulfide bond (1, 2). Apo(a) shares structural homology with plasminogen but lacks several functional domains (3). The apo(a) component contains 10 distinct kringle IV subtypes (KIV-1 through KIV-10), a kringle V domain, and an inactive protease-like domain (1–3). The KIV-2 domain exhibits marked copy-number variation that substantially influences both the molecular size of apo(a) and circulating Lp(a) levels (1–3). Figure 1 illustrates the structural features of Lp(a). The size of the apo(a) isoform reflects the KIV-2 copy number and is inversely related to plasma Lp(a) concentrations; individuals with fewer KIV-2 repeats produce a smaller apo(a) isoform that is more efficiently secreted, resulting in higher circulating Lp(a) levels (4).

## 2 Pathogenic mechanisms of Lp(a) in cardiovascular disease

Lp(a) is a key mediator of atherosclerotic cardiovascular disease through three principal pathogenic pathways. First, Lp(a) exerts a prothrombotic effect, largely attributable to the structural homology between apo(a) and plasminogen, which interferes with fibrinolysis and promotes thrombus formation (5). Second, Lp(a) is proatherogenic, as its LDL-like moiety facilitates cholesterol deposition within the arterial intima (5). Third, Lp(a) carries OxPLs on both the LDL-like moiety and kringle V of apo(a), serving as a major plasma carrier of OxPLs (1–3, 5, 6). These oxidized lipids trigger vascular inflammation, promote monocyte recruitment, accelerate atherogenesis, and contribute to plaque instability (5). On a molar basis, Lp(a) exhibits approximately 5- to 6-fold greater atherogenic potency than LDL, primarily due to its OxPL enrichment and pronounced proinflammatory potential (7).

## 3 Lipoprotein(a) as an independent risk factor for cardiovascular and valvular disease

The Copenhagen General Population Study demonstrated a progressive, concentration-dependent association between circulating Lp(a) levels and cardiovascular risk. Among approximately 70,000 participants followed for a median of 7.4 years, individuals with Lp(a) concentrations ≥50 mg/dL (>105 nmol/L) had a markedly higher incidence of major cardiovascular events than those with levels <10 mg/dL. This association persisted even among participants with well-controlled LDL-cholesterol concentrations (8).

Despite substantial LDL-cholesterol reduction achieved with rosuvastatin in the ASTRONOMER trial, no attenuation in the progression of aortic valve disease was observed (9). This finding indicates that Lp(a) functions as an LDL-independent predictor of calcific aortic valve disease progression (5). Participants with elevated baseline Lp(a) concentrations (>58.5 mg/dL) exhibited a significantly greater annual increase in aortic valve stenosis severity (*p* = 0.005) and an approximately 2-fold higher risk of aortic valve replacement or death compared with those in the lower tertiles (HR = 2.0; 95% CI: 1.1 to 3.7; *p* = 0.02) (5). Moreover, individuals with elevated Lp(a) concentrations (≥50 mg/dL) and high coronary artery calcium (CAC) scores (≥100 Agatston units) had a markedly greater 10-year ASCVD incidence compared with those with low Lp(a) and zero CAC (adjusted HR: 4.71; 95% CI: 3.01 to 7.40).

## 4 Epidemiology and genetic determinants of Lp(a)

Elevated Lp(a) is among the most common inherited lipid disorders. It is estimated that more than 1.4 billion people worldwide—approximately one-fifth of the global population—have Lp(a) concentrations above 125 nmol/L (50 mg/dL) (10). In the United States, approximately 35% of individuals have Lp(a) concentrations above 75 nmol/L (30 mg/dL) (11). Importantly, the atherothrombotic effects of Lp(a) may manifest at concentrations above 75 nmol/L (30 mg/dL) (10).

Plasma Lp(a) concentrations are primarily genetically determined, with more than 90% of interindividual variability explained by polymorphisms within the LPA gene (4, 12). Differences in KIV-2 repeat number determine apo(a) isoform size and drive most of the variation in circulating Lp(a) (4, 12). Therefore, substantial differences in Lp(a) distribution exist among ethnic groups, largely due to population-level variation in LPA allele frequencies (8, 10, 13, 14).

In the UK Biobank cohort, median Lp(a) concentrations were highest among Black participants (75 nmol/L), followed by South Asian (31 nmol/L), White (19 nmol/L), and Chinese (16 nmol/L) individuals (15). Although these ethnic disparities are well documented, current guidelines adopt a unified approach. The 2018 AHA/ACC Multisociety Cholesterol Guideline considers Lp(a) concentrations above 125 nmol/L (50 mg/dL) as a risk-enhancing factor in primary prevention (16).

## 5 Clinical assessment of Lp(a) in cardiovascular risk stratification

The epidemiologic and genetic characteristics of Lp(a) make measurements essential to identify individuals at increased cardiovascular risk. Plasma Lp(a) concentrations typically peak by approximately 5 years of age and remain relatively stable throughout life (4, 12). Therefore, most clinical guidelines recommend at least one lifetime measurement for clinical assessment and cardiovascular risk stratification (1, 8, 16–19). Nonetheless, certain non-genetic determinants can influence circulating Lp(a) concentrations. These include environmental, physiological, and pathological conditions such as aging, sex differences, menopause, inflammation, and kidney or liver disease (4, 8, 20).

Reassessment may be warranted in selected patient populations, particularly those at intermediate cardiovascular risk (8). For example, changes related to these non-genetic determinants may increase Lp(a) concentrations, potentially shifting an individual from an intermediate- to a higher-risk category. Therefore, measuring Lp(a) remains clinically important for cardiovascular risk assessment and for guiding the intensity of preventive interventions (1).

## 6 Underutilization of Lp(a) testing in clinical practice

Real-world data indicate that Lp(a) testing remains markedly underutilized in clinical practice (21–24). In an analysis of more than 5.5 million adults across six University of California health systems from 2012 to 2021, only 0.3% of all adults underwent Lp(a) testing (21). Even among higher-risk populations, testing was performed in only 3% of individuals with a family history of cardiovascular disease and in fewer than 4% of those with a personal history of cardiovascular disease (21). Recent data also show that fewer than 1% of adults in the United States have ever undergone Lp(a) measurement, with substantially lower testing rates among non-Hispanic Black individuals (OR: 0.68; 95% CI: 0.58 to 0.81) (22).

## 7 Analytical challenges in Lp(a) quantification

Accurate assessment of Lp(a) requires direct measurement, as standard lipid panels do not differentiate cholesterol derived from LDL particles from that transported by Lp(a) (25). Other circulating biomarkers, including apolipoprotein B and C-reactive protein, also do not typically capture the unique proatherogenic and procalcific properties of Lp(a) and are therefore inadequate substitutes for its measurement (26, 27).

The quantification of Lp(a) concentrations can be performed using either mass-based or particle-based assays (1). Mass assays report Lp(a) levels in milligrams per deciliter (mg/dL) and measure the total protein-lipid mass of circulating particles. Although mass assays are widely used in clinical laboratories, they are substantially influenced by apo(a) isoform size heterogeneity, which limits accuracy when estimating the true particle number (5, 28).

In contrast, particle-based assays quantify Lp(a) in nanomoles per liter (nmol/L), representing the molar concentration of Lp(a) particles rather than their aggregate mass. This approach is considered analytically superior, as it provides a more accurate estimation of particle burden and associated cardiovascular risk (5, 28). Nevertheless, particle-based assays remain less commonly available for routine use due to ongoing standardization challenges.

While direct conversions between mg/dL and nmol/L measurements are not scientifically valid, approximate conversions are sometimes used in clinical and research contexts to facilitate interpretation. Such estimates should be interpreted cautiously, given the inherent analytical imprecision.

## 8 Clinical management of patients with elevated lipoprotein(a)

The management of elevated Lp(a) requires a comprehensive approach that combines precise cardiovascular risk assessment, lifestyle optimization, and aggressive control of modifiable risk factors (1). In the primary prevention setting, Lp(a) measurement serves as an adjunct to conventional ASCVD risk estimation, particularly in individuals with a family history of premature ASCVD or elevated CAC scores (16). Although Lp(a) is currently considered a risk-enhancing marker, it is anticipated to become a therapeutic target as ongoing trials of Lp(a)-lowering agents report their outcomes. In patients with established ASCVD, elevated Lp(a) highlights the need for aggressive residual-risk reduction as part of secondary prevention (16).

To date, no pharmacologic agent has been approved specifically for the reduction of Lp(a) concentrations. The effects of existing lipid-lowering therapies on Lp(a) are variable and generally insufficient to meaningfully reduce Lp(a)-associated cardiovascular risk. Table 1 summarizes the effects of currently available lipid-lowering agents on circulating Lp(a) levels. Until targeted therapies become available, prevention management should emphasize comprehensive lifestyle interventions alongside optimal management of coexisting cardiometabolic conditions such as dyslipidemia, hypertension, and diabetes mellitus.

**Table 1:** Effects of available lipid-lowering therapies on lipoprotein(a) and ASCVD risk.

Therapy	Effect on Lp(a)	Effect on ASCVD risk
Statins	Increase (10–20%) (1, 36)	Reduce ASCVD events through LDL-C and apoB-containing atherogenic lipoproteins; the benefit is not mediated by Lp(a) (36). Statin therapy increases Lp(a) possibly through upregulation of hepatic LPA gene expression and enhanced apo(a) synthesis (36)
PCSK9 inhibitors (Alirocumab, Evolocumab)	Decrease (20–30%) (37, 38)	Alirocumab and evolocumab reduce MACE, with greater benefit observed in patients with higher baseline Lp(a) (37, 38). In ODYSSEY Outcomes, alirocumab reduced the risk of major events by 25% among individuals in the 75th percentile of Lp(a). In FOURIER, evolocumab reduced these events by 23% in participants above the median Lp(a) compared with 7% in those below. These findings suggest added cardiovascular protection potentially mediated through attenuation of Lp(a)-associated atherothrombotic risk
Inclisiran	Decrease (20–25%) (39)	Definitive cardiovascular outcome data are awaited from the ORION-4 and VICTORION-2 PREVENT (secondary prevention) and VICTORION-1 PREVENT (primary prevention in high-risk individuals) trials (40–42)
Niacin	Decrease (≈25%) (43, 44)	No evidence of ASCVD event reduction in AIM-HIGH or HPS2-THRIVE (43, 44)
Lipoprotein apheresis	Marked decrease (≈70%) (45)	Currently, the only approved therapy shown to lower Lp(a) and reduce cardiovascular risk (8, 18). Substantial reductions in cardiovascular event rates were observed within the first year of treatment, with benefits sustained for up to seven years. MACE and MANCE were reduced by 83–86% and 50–64%, respectively (45). However, the procedure is invasive, requires frequent sessions, and is associated with adverse effects such as bleeding related to heparin use, vascular access complications, and high treatment costs, limiting its practicality
Ezetimibe	Modest decrease (5–7%) (46); minimal/ no effects when combined with statin	Reduces ASCVD events primarily through LDL-C lowering (47)
Bempedoic acid	Minimal/inconsistent (−2 to +2%) (48, 49)	Reduces ASCVD events primarily through LDL-C lowering. Evidence from the CLEAR Outcomes trial demonstrated a 13% reduction in events among statin-intolerant patients (50)
Omega-3 fatty acids	Minimal/no effect (1)	Icosapent ethyl has demonstrated ASCVD risk reduction in certain patients with elevated triglyceride levels, but this benefit is not mediated by Lp(a)
Lomitapide/Evinacumab	Modest decrease (5–13%) (51, 52)	Approved for HoFH. Both agents achieve modest Lp(a) reductions (lomitapide by approximately 13% and evinacumab by approximately 5%), although evidence remains limited, and their use is restricted by their narrow therapeutic scope, high cost, and specialized administration requirements

apoB, apolipoprotein B; ASCVD, atherosclerotic cardiovascular disease; HoFH, homozygous familial hypercholesterolemia; LDL-C, low-density lipoprotein cholesterol; Lp(a), lipoprotein(a); MACEs, major adverse cardiovascular events; MANCEs, major adverse non-coronary events; PCSK9, proprotein convertase subtilisin/kexin type 9.

Early identification of individuals with elevated Lp(a) enables the timely implementation of preventive strategies that may mitigate future cardiovascular risk. Management should incorporate shared decision-making, particularly when considering cascade screening of first-degree relatives. Data from the UK Biobank cohort demonstrated a strong familial association with elevated Lp(a) levels, with elevated concentrations observed in more than 40% of first-degree and approximately 30% of second-degree relatives (29).

## 9 Emerging Lp(a)-lowering therapies

**Figure 2:**
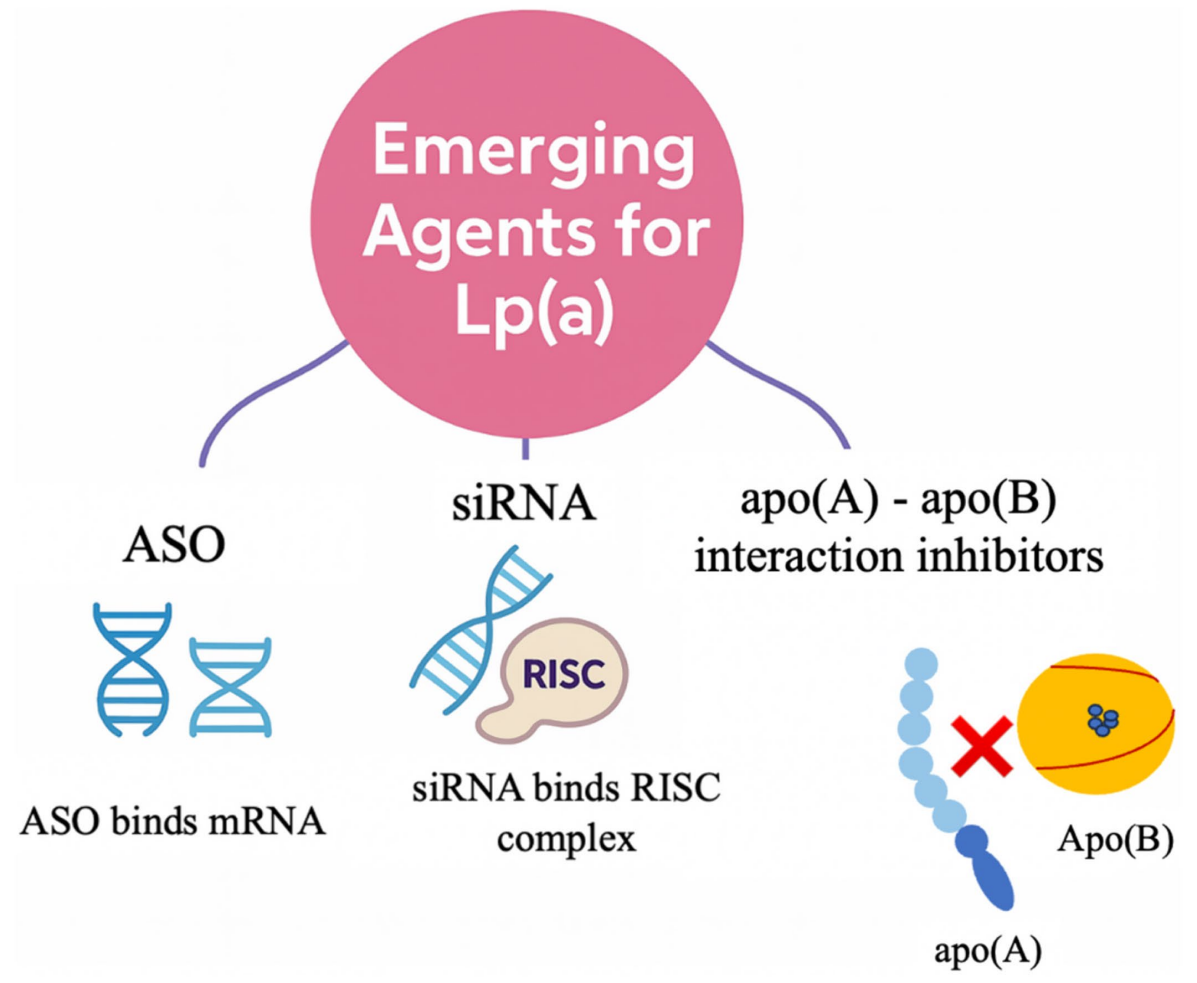
Emerging therapeutic approaches targeting lipoprotein(a). apo(a), apolipoprotein(a); apo(B), apolipoprotein B; ASO, Antisense oligonucleotide; RISC, RNA-induced silencing complex; siRNAs, Small interfering RNAs; Lp(a), lipoprotein(a).

Emerging therapies targeting Lp(a) act through three main mechanisms of action (Figure 2) (30). Antisense oligonucleotides (ASO), such as pelacarsen, consist of single-stranded nucleotides conjugated to N-acetylgalactosamine (GalNAc), which facilitates targeted delivery to hepatocytes via the asialoglycoprotein (ASGPR) receptors (1). Once internalized, these agents hybridize with LPA messenger RNA to form a duplex that recruits RNase H, leading to selective cleavage of the mRNA strand and inhibition of apo(a) protein synthesis (1).

Small interfering RNAs (siRNAs), including olpasiran, lepodisiran, and zerlasiran, follow a complementary pathway. These double-stranded, GalNAc-conjugated molecules are processed intracellularly, where the antisense strand is incorporated into the RNA-induced silencing complex (RISC), which subsequently guides it to LPA mRNA for cleavage and degradation, thereby silencing apo(a) production (1).

In addition to these gene-silencing strategies, muvalaplin introduces a third mechanism by disrupting Lp(a) particle assembly. As the first oral small-molecule Lp(a) inhibitor, muvalaplin prevents the binding of apo(a) to apo(B), thereby blocking the formation of the mature Lp(a) complex (30).

These therapeutic approaches have demonstrated substantial efficacy, reducing circulating Lp(a) concentrations by approximately 80–90%, with some regimens achieving near-complete suppression (Table 2). However, whether these biochemical reductions will translate into meaningful reductions in cardiovascular events remains to be determined, pending results from large-scale outcome trials. Nevertheless, early-phase clinical studies have reported favorable safety profiles across all classes of these investigational agents. Mild injection site reactions were the most frequently observed adverse events. Long-term use data are still needed to establish a more definitive understanding of the overall safety profile of these emerging therapies.

**Table 2:** Emerging pharmacological agents targeting Lp(a) reduction.

Drug/class	Key trial(s)	Phase	Key findings/details
Pelacarsen (TQJ230) ASO	AKCEA-APO(a)-LRx (35)	Phase 2 completed	Randomized, double-blind, placebo-controlled, dose-ranging trial in 286 patients with CVD and Lp(a) ≥ 60 mg/dL (150 nmol/L). Showed dose-dependent Lp(a) reduction of 35–80% (mean −80% with 20 mg weekly; *p* < 0.001). Also reduced oxidized phospholipids on apoB (−37 to −88%) and apo(a) (−28 to −70%). The treatment was well tolerated, with injection-site pain reported as the most common adverse event (27%)
	Lp(a)HORIZON (NCT04023552) (53)	Phase 3 ongoing (expected 2026)	Global, randomized, double-blind, placebo-controlled trial in 8,323 patients with established CVD and Lp(a) ≥ 70 mg/dL (175 nmol/L). Evaluates pelacarsen 80 mg SC monthly vs. placebo Primary endpoint: time to first MACE (CV death, non-fatal MI, non-fatal stroke, or urgent coronary revascularization)
	Lp(a)FRONTIERS CAVS (NCT05646381) (54)	Phase 2 ongoing (expected 2030)	Randomized, double-blind, placebo-controlled trial in 502 adults (aged 50–80 years) with Lp(a) ≥ 175 nmol/L and mild-to-moderate calcific aortic valve stenosis Primary endpoint: change in peak aortic jet velocity at 36 months
	ADD-VANTAGE (NCT06813911)	Phase 3 ongoing (expected 2027)	Multicenter, double-blind, placebo-controlled trial in 340 adults with ASCVD and Lp(a) ≥ 175 nmol/L on background inclisiran therapy Primary endpoint: change in log-transformed Lp(a) at 6 months
Olpasiran siRNA	OCEAN(a)-DOSE (34)	Phase 2 completed	Randomized, double-blind, placebo-controlled, dose-finding trial in 281 patients with ASCVD and Lp(a) > 150 nmol/L. Primary endpoint: percent change in Lp(a) at 36 weeks. All doses achieved significant reductions (*p* < 0.001), ranging from −70.5% to −101.1% at 75–225 mg every 12 weeks. The treatment was well tolerated, with injection-site pain as the most common adverse event (17%)
	OCEAN(a) (NCT05581303) (55)	Phase 3 ongoing (expected 2026)	Global, randomized, double-blind, placebo-controlled trial in 7,297 patients with ASCVD and Lp(a) ≥ 200 nmol/L. The study evaluates olpasiran 225 mg SC every 12 weeks vs. placebo with a median follow-up of 4 years Primary endpoint: time to CHD death, non-fatal MI, or urgent coronary revascularization
Lepodisiran (LY3819469) Extended-Duration siRNA	NCT04914546 (56)	Phase 1 completed	Randomized, double-blind, placebo-controlled, dose-ascending trial in 48 patients with Lp(a) ≥ 75 nmol/L. Single SC doses (4, 12, 32, 96, 304, or 608 mg). Produced dose-dependent reductions, with >90% lowering at the three highest doses that was sustained through 48 weeks (*p* < 0.001). Well tolerated; one serious adverse event unrelated to treatment, and mild injection-site reactions
	ALPACA (57)	Phase 2 completed	Randomized, double-blind, placebo-controlled, dose-ranging trial in 320 patients with elevated Lp(a) (median 254 nmol/L). Participants received lepodisiran 16, 96, or 400 mg SC at baseline and day 180. Lp(a) reductions were −41, −75%, and −94% (*p* < 0.001) sustained up to day 540 (−74% with 400 mg). The treatment was well tolerated, with mild injection-site reactions (≤12%) and no drug-related serious adverse events
	ACCLAIM-Lp(a) NCT06292013 (58)	Phase 3 ongoing (expected 2029)	Global, randomized, double-blind, placebo-controlled trial in 16,700 patients with ASCVD or at high risk for a first cardiovascular event and Lp(a) ≥ 175 nmol/L. The study evaluates lepodisiran administered subcutaneously versus placebo, with an estimated follow-up of approximately 4.5 years Primary endpoint: time to first MACE-4 event (CV death, non-fatal MI, non-fatal ischemic stroke, or urgent coronary revascularization)
Zerlasiran (SLN360) siRNA	NCT04606602 (59)	Phase 1 completed	Randomized, double-blind, placebo-controlled trial in 68 participants (32 healthy, 36 with ASCVD) and Lp(a) ≥ 150 nmol/L. Two SC doses (200, 300, or 450 mg) achieved 97–99% maximal Lp(a) reductions, sustained at 60–90% at 6 months. Single dose of 300 or 600 mg produced approximately a 30% reduction at 1 year. The treatment was well tolerated, with no serious adverse events reported
	NCT05537571 (60)	Phase 2 completed (results pending)	Global, randomized, double-blind, placebo-controlled trial in 180 participants aged 18–80 years with elevated Lp(a) ≥ 125 nmol/L and high risk of ASCVD. The study evaluates subcutaneous zerlasiran 300 mg every 16 weeks, 300 mg every 24 weeks, or 450 mg every 24 weeks versus placebo, with a 36-week treatment duration Primary endpoint: time-averaged percent change in Lp(a) from baseline to week 36
Muvalaplin (LY3473329) Oral, small molecule Lp(a) inhibitor	NCT04472676 (61)	Phase 1 completed	Randomized, double-blind, placebo-controlled trial in 114 healthy participants. Single doses (1–800 mg) and multiple daily doses (30–800 mg for 14 days) achieved placebo-adjusted Lp(a) reductions up to 65%, with similar effects at doses ≥100 mg. The treatment was well tolerated, with only mild, transient adverse events and no serious safety concerns
	KRAKEN (62)	Phase 2 completed	Randomized, double-blind, placebo-controlled, dose-ranging trial in 233 patients aged ≥40 years with Lp(a) ≥ 175 nmol/L and either established ASCVD or risk-equivalent conditions (familial hypercholesterolemia or type 2 diabetes). Participants received oral muvalaplin 10, 60, or 240 mg daily for 12 weeks. Lp(a) reductions were 47.6, 81.7, and 85.8% by the intact Lp(a) assay and 40.4, 70.0, and 68.9% by apolipoprotein(a)-based assay (all *p* < 0.001). The treatment was well tolerated, with only mild, transient adverse events and no serious safety concerns

ASCVD, atherosclerotic cardiovascular disease; apo(a), apolipoprotein(a); apoB, apolipoprotein B; ASO, antisense oligonucleotide; CHD, coronary heart disease; CV, cardiovascular; CVD, cardiovascular disease; Lp(a), lipoprotein(a); MACE, major adverse cardiovascular events; MI, myocardial infarction; nmol/L, nanomoles per liter; SC, subcutaneous; siRNA, small interfering RNA.

## 10 Magnitude of Lp(a) reduction required for clinical benefits

Accumulating genetic evidence indicates that substantial absolute reductions in Lp(a) levels are necessary to achieve measurable cardiovascular benefits. A landmark Mendelian randomization analysis demonstrated a linear, dose-dependent association between genetically determined Lp(a) concentrations and the risk of coronary heart disease (CHD) (31). The study suggested that the cardiovascular benefit is proportional to the absolute magnitude of Lp(a) lowering rather than to relative percentage change. Each 10 mg/dL decrement in genetically predicted Lp(a) corresponded to an approximately 5.8% reduction in CHD odds (OR 0.94; 95% CI 0.93–0.95) (31).

Modeling of this relationship indicated that an absolute reduction of approximately 100 mg/dL would be required to achieve a CHD risk reduction comparable to lowering LDL-cholesterol by 38.67 mg/dL (1 mmol/L)—a degree of LDL-cholesterol lowering consistently associated with a 20–25% decrease in major cardiovascular events (32). Therefore, achieving meaningful cardiovascular benefit likely requires lowering Lp(a) by approximately 105–210 nmol/L (50–100 mg/dL) (7). This finding may provide a mechanistic rationale for the neutral outcomes observed in earlier trials of agents such as niacin, which reduced Lp(a) by 20–30% yet produced limited absolute changes and, consequently, no discernible Lp(a)-specific benefit.

## 11 Conclusions and future directions

Although novel Lp(a)-lowering agents have demonstrated marked efficacy, often achieving reductions of 80–90%, it remains uncertain whether these substantial biochemical effects will translate into proportional reductions in cardiovascular events. Additionally, these therapies have been shown to lower OxPLs and vascular inflammation, suggesting additive cardiovascular benefits beyond Lp(a) lowering (7, 33–35).

Ongoing cardiovascular outcome trials will be essential to determine whether achieving such profound decreases in Lp(a) can meaningfully modify residual cardiovascular risk and confirm the predictions derived from genetic modeling. If these trials confirm its clinical benefit, Lp(a)-targeted therapy may become available in routine practice within the next few years, shifting Lp(a) from a risk modifier to a treatable therapeutic target.
